# Supplementary figures and images for: Generalized linear mixed models can detect unimodal species-environment relationships
Source: PeerJ. 2013 Jul 9;1:e95. doi: 10.7717/peerj.95 (PMC3709111; doi:10.7717/peerj.95)

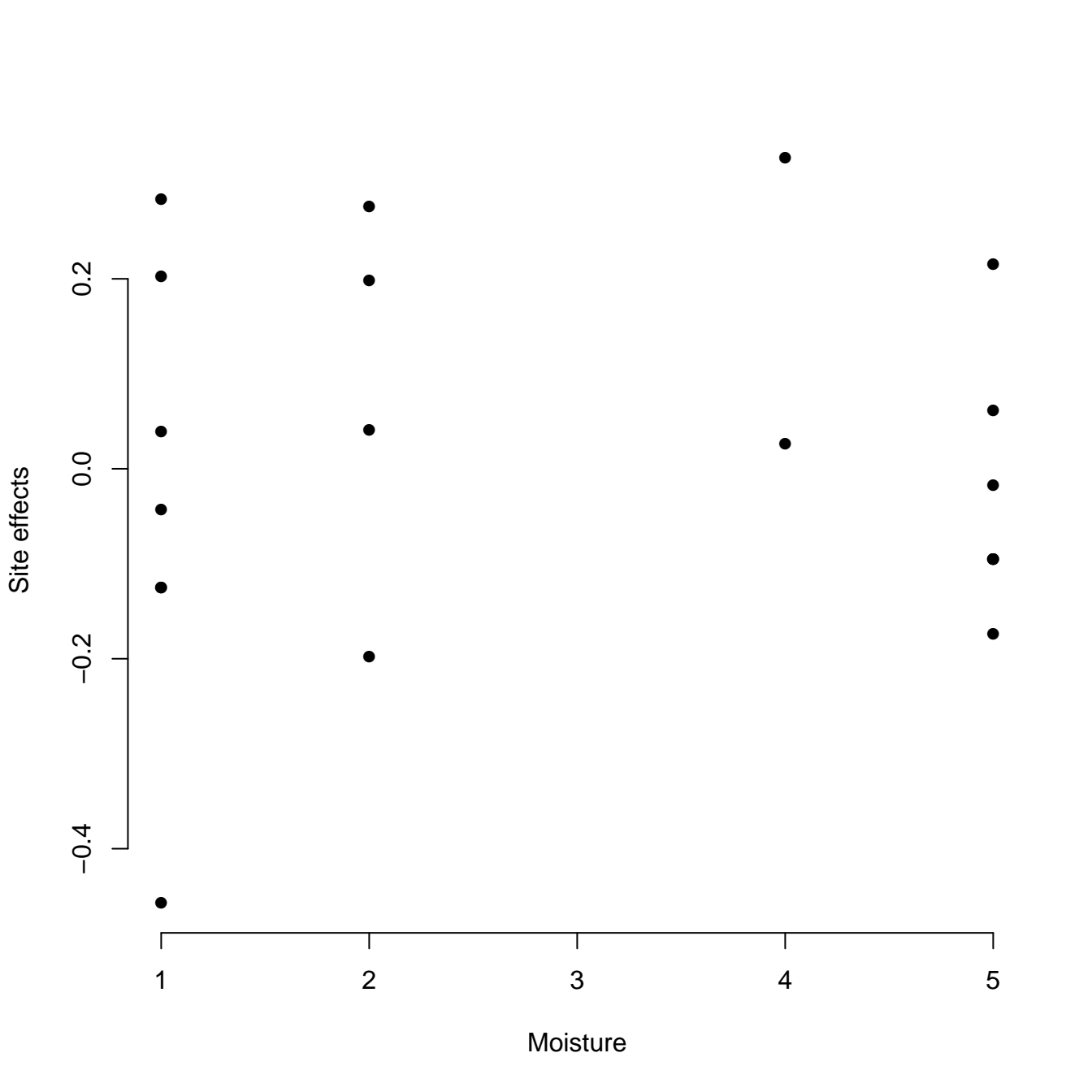

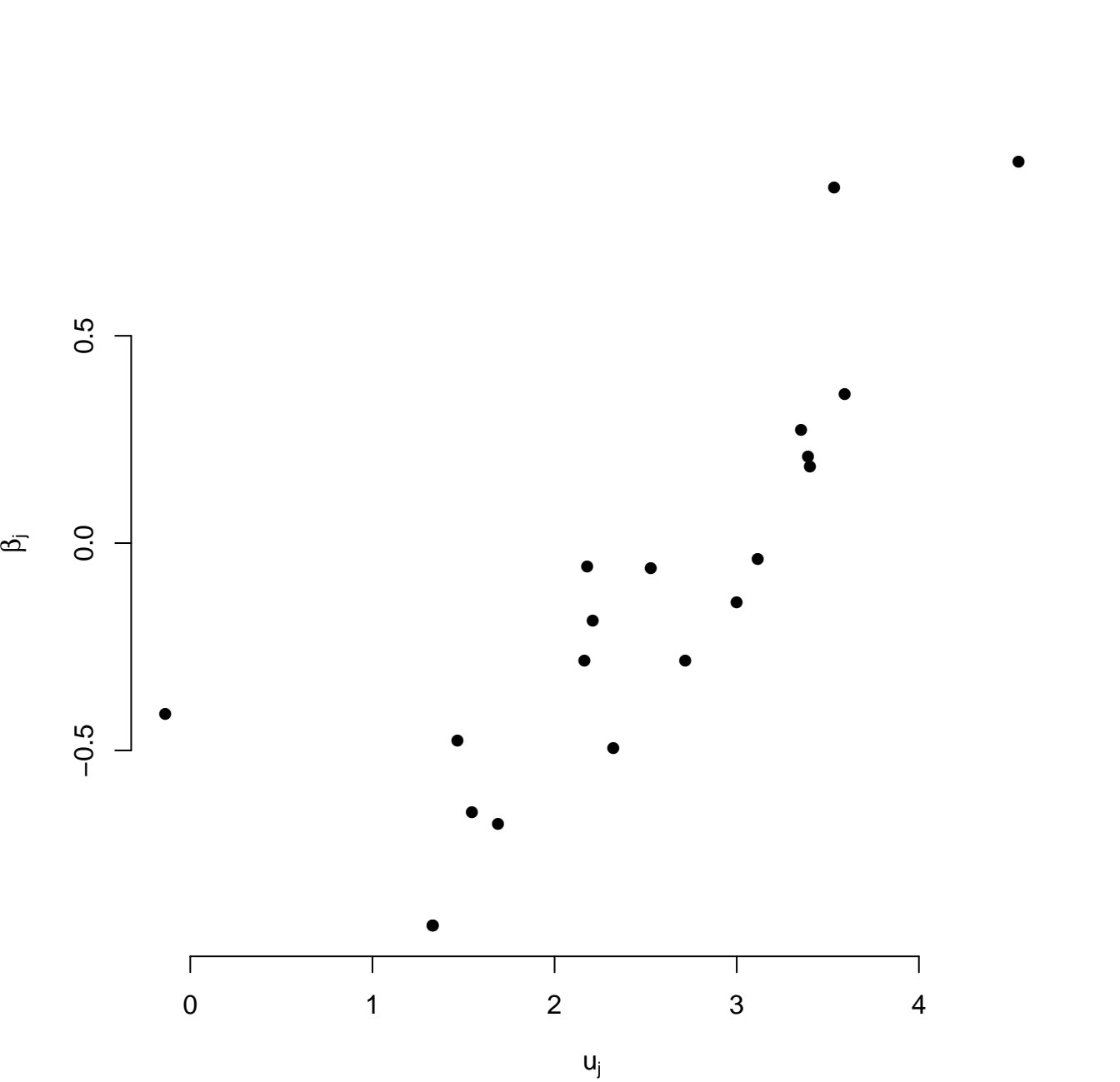

Supplement: Supplemental Information 1 — The zip file contains three files. The file “Rcode_with_example.r” is the R-script with R-function Test.Graph.unimodal and application to the dune data. The file “Rcode_with_example_output.txt” contains the output of the R-script and the file “Rcode_with_example_plot.pdf” the produced plot. [file peerj-01-95-s001.zip › Rcode_with_example_plot.pdf]
